# Knowledge, Attitudes, and Practices Toward Perinatal Depression Among Obstetric Healthcare Professionals at a Tertiary Care Center in Mumbai, India

**DOI:** 10.7759/cureus.104636

**Published:** 2026-03-03

**Authors:** Karubaki Utkalika, Himangi S Warke

**Affiliations:** 1 Department of Obstetrics and Gynaecology, Seth Gordhandas Sunderdas (GS) Medical College and King Edward Memorial (KEM) Hospital, Mumbai, IND

**Keywords:** attitudes and practices, knowledge, maternal mental health, obstetric healthcare professionals, screening

## Abstract

Background

Perinatal depression is a common mental health condition with substantial adverse consequences for maternal well-being and child development. In India, obstetric healthcare professionals are often the first and most consistent point of contact for women during the antenatal and postpartum periods. This places them in a pivotal position to identify and facilitate early intervention for perinatal depression. However, evidence regarding their knowledge, attitudes, and practices related to perinatal mental health remains limited.

Aim

The study aimed to assess the knowledge, attitudes, and practices related to perinatal depression among obstetric healthcare professionals in a tertiary care setting in Mumbai and to identify perceived barriers to routine screening and management.

Methods

A five-month cross-sectional, questionnaire-based study was conducted in the Department of Obstetrics and Gynecology at a tertiary care teaching hospital in Mumbai. Obstetric healthcare professionals, including interns, junior residents, senior residents, and consultants, were recruited using purposive sampling. A structured, self-administered, validated questionnaire assessed professional characteristics and knowledge, attitudes, and practices related to perinatal depression. Descriptive statistics were used to summarize participant characteristics and responses in the knowledge, attitude, and practice domains. Categorical variables were expressed as frequencies and percentages. Associations between categorical variables (e.g., professional designation, prior mental health training, and screening practices) were analyzed using the Chi-squared test of independence. A p-value of <0.05 was considered statistically significant.

Results

A total of 92 obstetric healthcare professionals participated. While 72 (78.3%) were aware of perinatal depression as a condition affecting women during pregnancy and the postpartum period, only 39 (42.4%) reported awareness of validated screening tools. Although attitudes toward perinatal mental health were largely positive, only 25 (27.2%) reported routinely screening women. Commonly identified barriers included lack of formal training, time constraints in busy clinical settings, limited access to mental health services, and unclear referral pathways. A significant knowledge-practice gap was observed, with higher screening practices among senior clinicians and those with prior mental health training (p < 0.001).

Conclusion

Despite adequate knowledge and favorable attitudes, routine screening for perinatal depression remains suboptimal. Targeted training, integration of standardized screening tools into routine obstetric care, and strengthened referral pathways are essential to improve early identification and management of perinatal depression.

## Introduction

Globally, increasing attention is being directed toward maternal mental health as an important public health concern. Perinatal mental illness (PMI) encompasses a range of mental health conditions occurring during pregnancy and the postpartum period. Although severe mental illnesses are relatively uncommon, common mental disorders (CMDs), particularly depression and anxiety, contribute substantially to maternal morbidity and mortality [1]. These conditions often remain underdiagnosed and undertreated, despite their profound impact on maternal functioning, quality of life, and caregiving capacity. Women in low- and middle-income countries (LMICs), including India, are disproportionately affected by perinatal mental disorders due to a convergence of psychosocial stressors such as socioeconomic deprivation, inadequate social support, and gender-based inequities [2]. Evidence consistently demonstrates that perinatal depression and anxiety are associated with adverse maternal and offspring outcomes, including impaired mother-infant bonding and poorer social-emotional, cognitive, language, motor, and adaptive behavioral development in children [3]. The burden of perinatal mental disorders in LMICs is considerable, with a pooled prevalence of approximately 18.6% [4]. In India, antenatal CMDs have been reported in nearly 21.8% of women, while postpartum depression affects approximately 22%, underscoring the magnitude of the problem within routine maternal health services [5,6]. Despite national and international initiatives such as mhGAP (Mental Health Gap Action Program) and task-sharing models such as the PRogramme for Improving Mental Health Care (PRIME) that trained maternity care providers, routine perinatal depression screening in obstetric settings in India remains limited. It is because of insufficient training, time constraints, stigma, and unclear referral pathways [7-9]. There remains a need for obstetrician-specific training programs that are tailored to women’s health and leverage obstetricians’ role as the first point of contact for antenatal and postnatal women [10,11]. Obstetric healthcare professionals, including interns, residents, and consultants, are often the primary and most consistent point of contact for women during the antenatal and postpartum periods, positioning them uniquely to facilitate early identification and referral for perinatal depression. Assessing their knowledge, attitudes, and practices (KAP) is therefore a critical step toward informing targeted training initiatives and strengthening health systems to better integrate perinatal mental health care into routine obstetric services [12]. Accordingly, the present study aimed to assess the KAP related to perinatal depression among obstetric healthcare professionals in a tertiary care setting in Mumbai and to identify perceived barriers to routine screening and management.

This study was previously presented as a meeting abstract at the XXV FIGO World Congress of Gynecology and Obstetrics on October 5, 2025, and the abstract was subsequently published in the supplementary issue of the International Journal of Gynecology & Obstetrics (Volume 171, Issue S2).

## Materials and methods

Study design and setting

A cross-sectional, questionnaire-based study was conducted in the Department of Obstetrics and Gynecology at Seth Gordhandas Sunderdas (GS) Medical College and King Edward Memorial (KEM) Hospital, a tertiary care teaching hospital in Mumbai, India. The study was conducted from September 2025 to February 2026.

Study participants and eligibility criteria

The study population comprised obstetric healthcare professionals actively involved in the care of pregnant and postpartum women. Clinical responsibility ranged from supervised participation to independent, comprehensive maternal care, indicating wide variation in clinical experience. Participants included interns, junior residents, senior residents, and consultants.

Inclusion criteria were medical professionals engaged in obstetric practice and willing to provide informed consent. Healthcare professionals not directly involved in obstetric care and those who declined participation were excluded.

Sample size and sampling technique

The sample size was calculated using Cochrane’s formula for finite populations, assuming a 95% confidence level and 5% margin of error, based on an estimated population of obstetric healthcare professionals at the study site. The final sample size was determined to be 92 participants. A purposive sampling technique was employed to ensure representation across different professional designations.

Study instrument

Data were collected using a structured, self-administered questionnaire designed to assess KAP related to perinatal depression (Appendix). The questionnaire consisted of four sections: (1) professional details, including age, designation, years of experience, and prior exposure to mental health training; (2) knowledge domain, assessing awareness of perinatal depression, risk factors, clinical features, screening tools, and management principles; (3) attitude domain, evaluating perceptions regarding importance of perinatal mental health, stigma, confidence in screening, and perceived responsibility in care; and (4) practice domain, assessing routine screening behaviors, referral practices, access to mental health services, and perceived barriers.

Validity of the questionnaire

Content validation of the questionnaire was performed by a multidisciplinary expert panel comprising two obstetricians, one psychiatrist, and two medical education experts. The tool was refined based on expert feedback to ensure clarity, relevance, and content adequacy prior to administration.

Data collection procedure

After obtaining written informed consent, the questionnaire was administered in person to eligible participants during working hours. Participation was voluntary and anonymous, and no personally identifiable information was collected.

Statistical analysis

Data were entered and analyzed using SPSS version 21.0 (IBM Corp., Armonk, NY, USA). Descriptive statistics were used to summarize participant characteristics and responses in the KAP domains. Categorical variables were expressed as frequencies and percentages. Associations between categorical variables (e.g., professional designation, prior mental health training, and screening practices) were analyzed using the Chi-squared test of independence. For 2 × 2 contingency tables, odds ratios (ORs) with 95% confidence intervals (CIs) were calculated to estimate the strength of association. A p-value of <0.05 was considered statistically significant.

Ethical considerations

The study was approved by the Institutional Ethics Committee of Seth GS Medical College and KEM Hospital. Participants were informed that participation was voluntary and confidential and would not affect their professional role. Informed consent was obtained prior to data collection.

## Results

Participant characteristics

A total of 92 obstetric healthcare professionals participated in the study, including interns, junior residents, senior residents, and consultants involved in antenatal and postnatal care. All participants reported regular clinical contact with pregnant and postpartum women. Detailed professional characteristics of the participants are presented in Table 1.

**Table 1:** Professional characteristics of obstetric healthcare professionals (n = 92) Distribution of study participants according to professional designation, years of obstetric experience, and clinical contact with antenatal and postnatal women. Data are presented as frequency (n) and percentage (%).

Variable	Frequency (n)	Percentage (%)
Designation		
Interns	36	39.1
Junior residents	34	37.0
Senior residents	10	10.9
Consultants	12	13.0
Total	92	100
Years of experience in obstetrics		
Less than 5 years	70	76.1
5-10 years	10	10.9
More than 10 years	12	13.0
Total	92	100
Regular clinical contact with antenatal/postnatal women		
Yes	92	100
No	0	0

Knowledge regarding perinatal depression

The KAP and perceived barriers related to perinatal depression are summarized in Table 2. Overall, 72 (78.3%) of the participants were aware of perinatal depression as a mental health condition affecting women during pregnancy and the postpartum period. Knowledge regarding risk factors and clinical features of perinatal depression was generally adequate. However, awareness of validated screening tools was comparatively lower, with only 39 (42.4%) of participants reporting awareness of validated screening instruments and considering them important for clinical practice. These findings indicate that while general awareness of perinatal depression was high, familiarity with formal screening approaches was limited. Screening practices differed significantly by professional designation, with higher screening rates observed among senior clinicians (χ² (3) = 16.94, p < 0.001), as presented in Table 3.

**Table 2:** Knowledge, attitudes, and practices (KAP) regarding perinatal depression among obstetric healthcare professionals (n = 92) Distribution of participants’ KAP and perceived barriers related to perinatal depression. Data are presented as frequency (n) and percentage (%) calculated using the total sample size (n = 92). EPDS: Edinburgh Postnatal Depression Scale

Domains	KAP questions	n (%)
Knowledge	Correctly identified global burden	65 (70.7)
	Correctly defined perinatal period	72 (78.3)
	Recognized hormonal role	60 (65.2)
	Identified key symptoms	74 (80.4)
	Identified risk factors	69 (75.0)
	Awareness of screening tools	39 (42.4)
	Identified EPDS as a common tool	34 (37.0)
	First-line treatment psychotherapy	68 (73.9)
Attitudes	Depression is a serious condition (agree/strongly agree)	86 (93.5)
	Screening is essential (agree/strongly agree)	82 (89.1)
	Impacts mother-infant bonding (agree/strongly agree)	84 (91.3)
	Depression is a personal weakness (disagree)	78 (84.8)
Practices	Always/often screen	25 (27.2)
	Refer to a mental health professional	70 (76.1)
	Use standardized tools	28 (30.4)

**Table 3:** Factors associated with routine screening for perinatal depression among obstetric healthcare professionals (n = 92) The Chi-squared test of independence was used to assess associations between categorical variables. p < 0.05 considered statistically significant; p < 0.001 considered highly statistically significant. * denotes statistically significant association. Odds ratios (ORs) with 95% confidence intervals (CIs) are reported for 2 × 2 comparisons only. Hyphens (-) indicate values not applicable.

Variable	Screen n (%)	Do not screen n (%)	χ² (df)	p-value	OR (95% CI)
Prior mental health training			18.99 (1)	<0.001*	12.34 (3.74-40.73)
Yes (n = 41)	21 (51.2%)	20 (48.8%)	-	-	-
No (n = 51)	4 (7.8%)	47 (92.2%)	-	-	-
Professional designation			16.94 (3)	<0.001*	-
Interns (n = 36)	4 (11.1%)	32 (88.9%)	-	-	-
Junior residents (n = 34)	8 (23.5%)	26 (76.5%)	-	-	-
Senior residents (n = 10)	6 (60.0%)	4 (40.0%)	-	-	-
Consultants (n = 12)	7 (58.3%)	5 (41.7%)	-	-	-

Attitude toward perinatal mental health

Participants demonstrated a predominantly positive attitude toward perinatal mental health care. Most respondents agreed that perinatal depression is a serious mental health condition and acknowledged its potential impact on maternal and infant well-being. A majority also endorsed the importance of identifying and addressing perinatal mental health concerns within obstetric practice. Overall, attitudinal responses reflected high perceived importance of perinatal mental health, despite variability in confidence regarding implementation.

Practices related to screening and management

**Figure 1:**
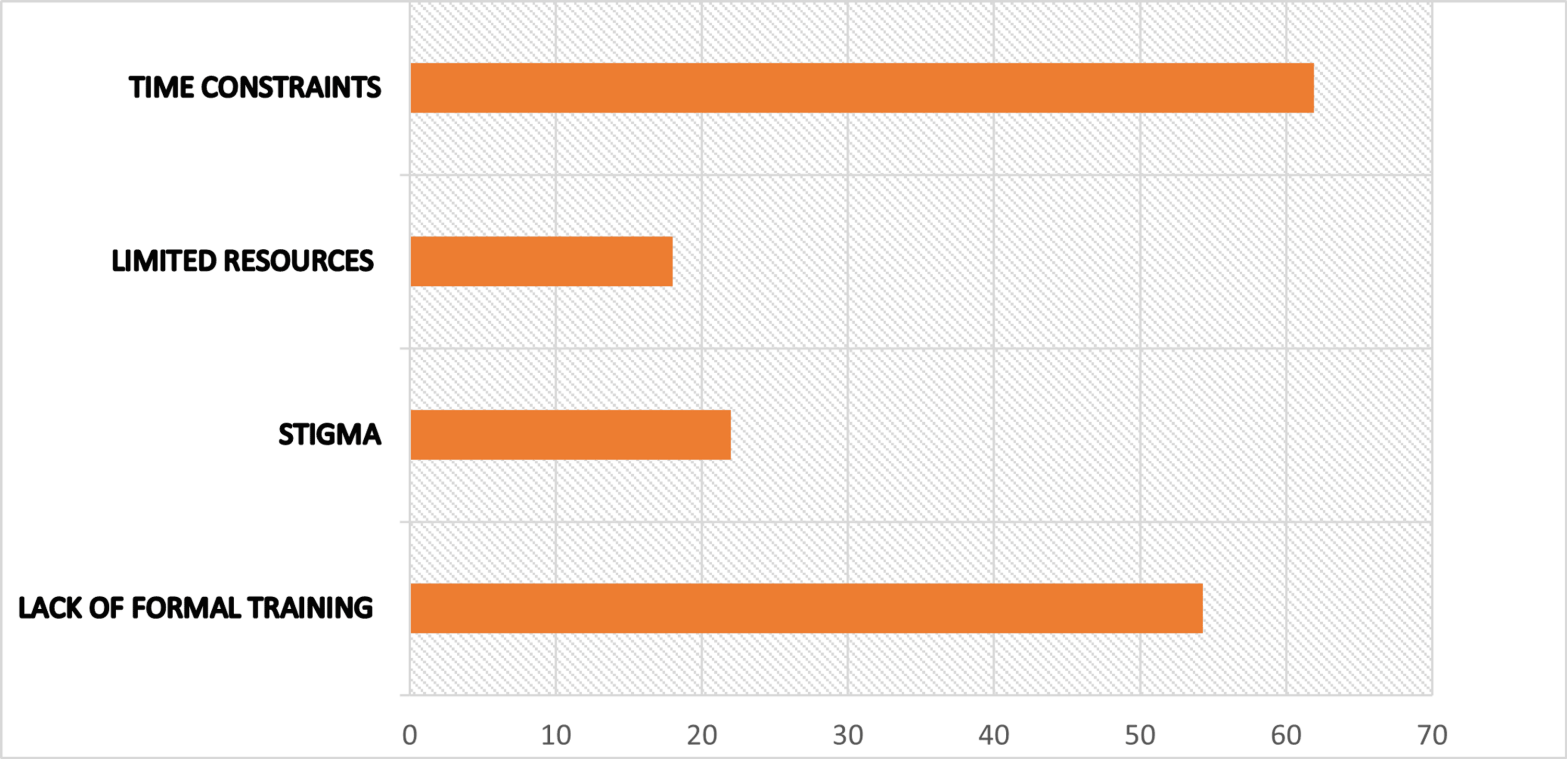
Main barriers to screening implementation

Despite relatively high awareness and positive attitudes, screening practices were suboptimal. Only 25 (27.2%) of participants reported routinely screening women for perinatal depression during antenatal or postnatal visits. When perinatal depression was suspected, referral to mental health professionals was commonly reported; however, standardized screening tools were infrequently used in routine clinical practice. The most frequently reported barriers to screening included lack of formal training, time constraints in busy outpatient settings, limited access to mental health services, and uncertainty regarding referral pathways (Figure 1).

Association between KAP

The findings revealed a clear knowledge-practice gap, wherein high awareness and favorable attitudes toward perinatal depression did not translate into routine screening practices. Participants with prior exposure to formal mental health training, including undergraduate psychiatry postings, postgraduate academic sessions, workshops, or continuing medical education (CME) programs on perinatal mental health and in-service sensitization, were significantly more likely to screen for perinatal depression than those without such training (χ² (1) = 18.99, p < 0.001) (Table 3). The odds of routine screening were 12.34 times higher among trained professionals (OR = 12.34; 95% CI: 3.74-40.73).

**Figure 2:**
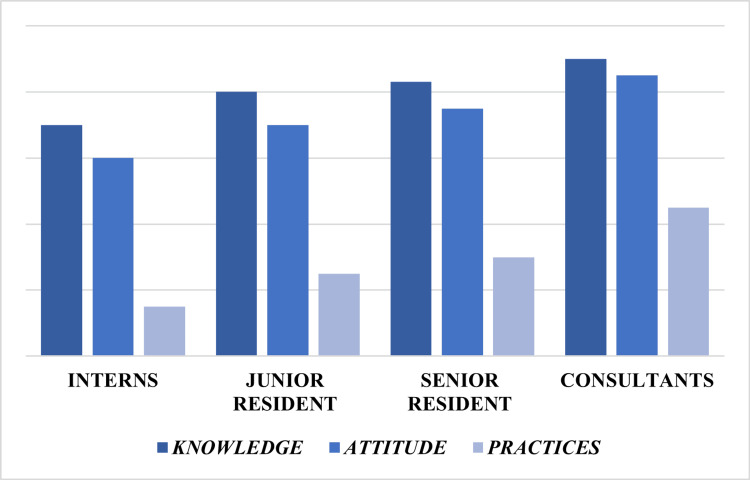
Subgroup differences in knowledge, attitudes, and practices

Screening practices also varied significantly by professional designation (χ² (3) = 16.94, p < 0.001), with higher screening rates observed among senior residents and consultants compared to interns and junior residents. Subgroup differences in knowledge and screening practices are depicted in Figure 2.

## Discussion

Addressing a critical evidence gap, this study evaluates KAP related to perinatal depression among obstetric healthcare professionals in a tertiary care setting in India. The findings demonstrate that while most participants possessed moderate to good knowledge of perinatal depression and expressed favorable attitudes toward its recognition and management, these did not consistently translate into routine screening and standardized clinical practices. Similar to earlier studies conducted among obstetricians and maternity care providers, the present study highlights a persistent gap between awareness and implementation of perinatal mental health care [12,13]. Perinatal depression is widely recognized as a significant contributor to maternal morbidity worldwide, with long-term consequences for both maternal functioning and child development [14,15]. Increasing global advocacy has emphasized that there is “no health without perinatal mental health,” reinforcing the need for integration within obstetric services [16,17]. Previous research has shown that negative or ambivalent attitudes among maternity care providers can influence both their understanding of maternal mental health issues and professional behavior, thereby reducing opportunities for early identification during the perinatal period. In the present study, although most participants acknowledged perinatal depression as a serious condition and endorsed the importance of screening, a proportion reported discomfort in initiating mental health-related discussions and limited confidence in assessing suicide risk [18]. These findings underscore the need for focused educational initiatives that address not only knowledge deficits but also attitudinal and confidence-related barriers among obstetric healthcare professionals. Continuous professional development and structured sensitization programs may be necessary to sustain favorable attitudes and normalize mental health discussions within obstetric practice [19]. Despite adequate knowledge and largely positive attitudes, screening practices were suboptimal, with only a minority of participants reporting routine screening for perinatal depression or regular use of validated tools. Similar gaps between knowledge and practice have been reported in previous studies among obstetric and primary care providers. Subgroup analysis in the present study revealed higher knowledge levels and screening practices among senior clinicians and those with prior exposure to mental health training; however, gaps in routine screening persisted across all professional groups. Time constraints in high-volume obstetric settings further limited opportunities for mental health assessment, with competing clinical priorities and limited consultation time acting as important practical barriers to routine screening. This suggests that experience alone is insufficient to ensure consistent implementation of screening practices. Evidence from earlier studies indicates that provider education, incorporation of standardized screening tools such as the Edinburgh Postnatal Depression Scale into routine workflows, and clear referral pathways can improve screening compliance [20-24]. Furthermore, untreated perinatal depression has been associated with adverse maternal and child developmental outcomes [25]. The heightened psychological vulnerability of women during periods of increased stress further emphasizes the importance of equipping obstetric healthcare professionals with the skills and systems required for early identification and timely referral of perinatal depression. Strengthening perinatal mental health integration within obstetric services is therefore essential to improve maternal and child health outcomes in the Indian context.

Strengths and limitations

This study has several strengths. To the best of our knowledge, it is among the few studies from India to comprehensively assess KAP related to perinatal depression among obstetric healthcare professionals. The inclusion of participants across different professional designations enhances the relevance of the findings to tertiary care obstetric settings. However, certain limitations must be acknowledged. The cross-sectional design precludes causal inferences. Data were based on self-reported practices, which may be subject to recall and social desirability bias. The study was conducted at a single tertiary care center, which may limit generalizability to other healthcare settings. Additionally, psychometric evaluation of the questionnaire was limited to content validation.

## Conclusions

The present study demonstrates that although obstetric healthcare professionals possess adequate knowledge and favorable attitudes toward perinatal depression, routine screening and standardized assessment practices remain limited. The observed knowledge-practice gap highlights the need for structured, obstetrician-specific training, integration of screening tools into routine obstetric workflows, and clear referral pathways to mental health services. Addressing these gaps may strengthen early identification and management of perinatal depression and improve maternal and child health outcomes.

## Appendices

Case Record Form

Study Title: Perinatal Depression: Knowledge, Attitude, and Practices Among Medical Professionals in Obstetrics in a Tertiary Care Setting

Case No.

Demographic Information

1. Years of experience in obstetrics:

☐ Less than 5 years   ☐ 5-10 years   ☐ More than 10 years

2. Designation:

☐ Intern   ☐ Junior resident   ☐ Senior resident   ☐ Consultant   ☐ Other: ___________

3. Regular clinical contact with antenatal and postnatal women

☐ Yes ☐ No

Knowledge

4. Which of the following best reflects the global burden of perinatal depression?

☐ It is a rare condition affecting fewer than 5% of women.

☐ It affects a significant proportion, approximately 10%-20% of women during the perinatal period.

☐ It affects only women with a past psychiatric history.

☐ It is mainly a concern in high-income countries with increased mental health awareness.

5. Which of the following best defines the period included in perinatal depression as per global and national maternal mental health frameworks?

☐ Depression occurring during pregnancy only

☐ Depression occurring up to 6 weeks after childbirth

☐ Depression occurring during both pregnancy and up to 12 months postpartum

☐ Depression that begins after stopping breastfeeding

6. What is the role of hormonal changes in the development of perinatal depression?

☐ Hormonal changes are the primary cause

☐ Hormonal changes contribute

☐ Hormonal changes have no role

☐ Hormonal changes are a result

7. Which of the following is/are common symptoms of perinatal depression? (Select all that apply)

☐ Persistent sadness or low mood

☐ Excessive worry or anxiety

☐ Changes in sleep or appetite

☐ Loss of interest or pleasure in usual activities

☐ Irritability or feelings of guilt

8. Which somatic symptom is common in both antenatal depression and pregnancy, making diagnosis difficult? (Select all that apply)

☐ Nausea and vomiting

☐ Chest pain

☐ Weight loss

☐ Fever

9. Which of the following are established risk factors for perinatal depression? (Select all that apply)

☐ Personal or family history of depression or anxiety

☐ Lack of social or emotional support

☐ Hormonal and neurochemical changes during pregnancy/postpartum

☐ Pregnancy complications (e.g., pre-eclampsia and gestational diabetes)

☐ Intimate partner violence or marital conflict

10. Which factor is/are most predictive of postpartum depression?

☐ Rural residence

☐ Lack of partner or family support

☐ Primigravida status

☐ Breastfeeding difficulties

11. A mother reports difficulty bonding with her newborn, despite having no medical complications. Which condition is most likely?

☐ Baby blues

☐ Postpartum psychosis

☐ Postpartum depression

☐ Postnatal anxiety

12. Which of the following tools are available for screening or diagnosing perinatal depression? (Select all that apply)

☐ Edinburgh Postnatal Depression Scale (EPDS)

☐ Patient Health Questionnaire-9 (PHQ-9)

☐ Beck Depression Inventory (BDI)

☐ Generalized Anxiety Disorder-7 (GAD-7)

☐ Postpartum Depression Screening Scale (PDSS)

☐ Hamilton Depression Rating Scale (HDRS)

13. What is the most commonly used screening tool for perinatal depression?

☐ Edinburgh Postnatal Depression Scale (EPDS)

☐ Patient Health Questionnaire-9 (PHQ-9)

☐ Beck Depression Inventory (BDI)

☐ Other (specify)

14. What is the recommended screening interval for perinatal depression during pregnancy?

☐ Once during pregnancy

☐ Screening is not needed unless symptoms appear

☐ At least once in each trimester or during routine antenatal visits

☐ Only if the woman has a psychiatric history

15. Which of the following is a barrier to diagnosing perinatal depression? (Select all that apply)

☐ Lack of training

☐ Limited access to mental health resources

☐ Stigma

☐ Time constraints

16. What is the recommended first-line treatment for women with mild perinatal depression according to current clinical guidelines?

☐ Antidepressant medication

☐ Psychotherapy (e.g., CBT and interpersonal therapy)

☐ Lifestyle modifications alone

☐ Combination of psychotherapy and medication

17. Which of the following classes of antidepressant medications are commonly used to treat perinatal depression? (Select all that apply)

☐ SSRIs: selective serotonin reuptake inhibitors

☐ SNRIs: serotonin-norepinephrine reuptake inhibitors

☐ TCAs: tricyclic antidepressants

☐ MAOIs: monoamine oxidase inhibitors

☐ NDRIs: norepinephrine-dopamine reuptake inhibitors

18. What is the recommended approach to managing perinatal depression in breastfeeding women?

☐ Avoid medication

☐ Use with caution

☐ Prioritize breastfeeding

☐ Prioritize treatment

19. What is the role of social support in the management of perinatal depression?

☐ Essential

☐ Helpful but not essential

☐ No role

☐ Can exacerbate depression

20. Which of the following are potential complications of untreated perinatal depression affecting the infant? (Select all that apply)

☐ Increased risk of infant mortality or low birth weight

☐ Poor maternal-infant bonding

☐ Impaired cognitive, emotional, or behavioral development in the child

☐ Increased risk of preterm birth

21. Which of the following is a benefit of screening for perinatal depression? (Select all that apply)

☐ Early detection and timely treatment

☐ Reduced risk of maternal and neonatal complications

☐ Improved maternal-infant bonding and caregiving

☐ Improved overall maternal mental health and quality of life

Attitude

Instructions: Rate your level of agreement with each statement using the following scale (mark the appropriate option):

1 = Strongly disagree   2 = Disagree   3 = Neutral   4 = Agree   5 = Strongly agree

22. Perinatal mental health receives inadequate emphasis in current obstetric training and clinical practice.

☐ Strongly disagree

☐ Disagree

☐ Neutral

☐ Agree

☐ Strongly agree

23. Perinatal depression is a serious mental health condition.

☐ Strongly disagree

☐ Disagree

☐ Neutral

☐ Agree

☐ Strongly agree

24. Perinatal depression can significantly affect the mother-infant relationship.

☐ Strongly disagree

☐ Disagree

☐ Neutral

☐ Agree

☐ Strongly agree

25. Routine screening for perinatal depression is essential for providing adequate care to pregnant and postpartum women.

☐ Strongly disagree

☐ Disagree

☐ Neutral

☐ Agree

☐ Strongly agree

26. Perinatal depression reflects a personal weakness rather than a medical condition.

☐ Strongly disagree

☐ Disagree

☐ Neutral

☐ Agree

☐ Strongly agree

27. Women with perinatal depression generally require hospitalization for treatment.

☐ Strongly disagree

☐ Disagree

☐ Neutral

☐ Agree

☐ Strongly agree

28. How often do you screen for perinatal depression in your patients?

☐ Always

☐ Often

☐ Sometimes

☐ Rarely

☐ Never

29. What do you usually do when you suspect a patient has perinatal depression?

☐ Refer to a mental health professional

☐ Prescribe antidepressant medication

☐ Provide counseling and support

☐ Other (please specify)

30. Do you have access to mental health resources for patients with perinatal depression?

☐ Yes

☐ No

☐ Maybe

31. How confident do you feel in managing perinatal depression?

☐ Very confident

☐ Somewhat confident

☐ Not very confident

☐ Not at all confident

32. What is your usual approach to treating perinatal depression?

☐ Medication only

☐ Psychotherapy only

☐ Combination of medication and psychotherapy

☐ Other (please specify)

33. Do you involve family members or support persons in the care of patients with perinatal depression?

☐ Yes

☐ No

☐ Maybe

34. How often do you follow up with patients who have been diagnosed with perinatal depression?

☐ At every prenatal or postpartum visit

☐ At some prenatal or postpartum visits

☐ Only when symptoms worsen

☐ Rarely or never

35. Have you received any training on perinatal depression?

☐ Yes

☐ No

☐ Maybe
